# Andrographolide Ameliorates Liver Fibrosis in Mice: Involvement of TLR4/NF-*κ*B and TGF-*β*1/Smad2 Signaling Pathways

**DOI:** 10.1155/2018/7808656

**Published:** 2018-03-18

**Authors:** Liteng Lin, Rui Li, Mingyue Cai, Jingjun Huang, Wensou Huang, Yongjian Guo, Liuhong Yang, Guizhi Yang, Tian Lan, Kangshun Zhu

**Affiliations:** ^1^Department of Minimally Invasive Interventional Radiology, and Department of Radiology, The Second Affiliated Hospital of Guangzhou Medical University, Guangzhou, China; ^2^Guangdong Pharmaceutical University, Guangzhou, China; ^3^PCFM Lab of Ministry of Education, School of Materials Science and Engineering, Sun Yat-sen University, Guangzhou, China

## Abstract

Liver fibrosis is characterized by activated hepatic stellate cells (HSC) and extracellular matrix accumulation. Blocking the activation of HSC and the inflammation response are two major effective therapeutic strategies for liver fibrosis. In addition to the long history of using andrographolide (Andro) for inflammatory disorders, we aimed at elucidating the pharmacological effects and potential mechanism of Andro on liver fibrosis. In this study, liver fibrosis was induced by carbon tetrachloride (CCl_4_) and the mice were intraperitoneally injected with Andro for 6 weeks. HSC cell line (LX-2) and primary HSC were also treated with Andro in vitro. Treatment of CCl_4_-induced mice with Andro decreased the levels of alanine aminotransferase (ALT) and aspartate aminotransferase (AST), Sirius red staining as well as the expression of *α* smooth muscle actin (*α*-SMA) and transforming growth factor- (TGF-) *β*1. Furthermore, the expression of Toll-like receptor (TLR)4 and NF-*κ*B p50 was also inhibited by Andro. Additionally, in vitro data confirmed that Andro treatment not only attenuated the expression of profibrotic and proinflammatory factors but also blocked the TGF-*β*1/Smad2 and TLR4/NF-*κ*B p50 pathways. These results demonstrate that Andro prevents liver inflammation and fibrosis, which is in correlation with the inhibition of the TGF-*β*1/Smad2 and TLR4/NF-*κ*B p50 pathways, highlighting Andro as a potential therapeutic strategy for liver fibrosis.

## 1. Introduction

Liver fibrosis is a reversible wound healing response which results from chronic liver injury in various chronic hepatic diseases, including hepatitis B and C, alcoholic liver disease, and nonalcoholic steatohepatitis [1, 2]. Advanced liver fibrosis might progress into irreversible cirrhosis, which is the leading cause of liver-related mortality worldwide [3]. Upon the activation of hepatic stellate cells (HSC) by various stimuli, such as transforming growth factor- (TGF-) *β*, quiescent HSC transdifferentiate into myofibroblasts and then play a key role in the pathogenesis of hepatic fibrosis by producing extracellular matrix (ECM) proteins [4].

The transforming growth factor-*β*1 (TGF-*β*1) has been well demonstrated to be the key cytokine during fibrogenesis. It plays a vital role in transforming quiescent HSC into fibrogenic myofibroblasts by stimulating the synthesis of ECM as well as inhibiting their degradation [5]. The TGF-*β*1 exerts its fibrogenic activities through the Smad signaling pathways, mainly the type I receptor-mediated phosphorylation of Smad2 and Smad3 [6]. The inhibition of TGF-*β*1 through both Smad2 and Smad3 significantly ameliorated liver fibrosis in several fibrotic animal models [7, 8]. Besides, TLR4 expressed on activated HSC enhanced TGF-*β*1-mediated HSC activation and ECM production [9]. TLRs belong to a highly conserved family of receptors that recognize pathogen-associated molecular patterns, which link inflammatory responses to inflammatory stimuli and allow the host to detect microbial infection [10]. TLR4 as a receptor for bacterial lipopolysaccharide (LPS), a well-known inducer of inflammation, is a primary mechanism by which HSC are activated during liver injury and fibrosis [11]. It can trigger the rapid activation of nuclear factor *κ*B (NF-*κ*B) via the myeloid differentiation factor 88- (MyD88-) dependent pathway, which upregulates the profibrogenic cytokines including TGF-*β*1 [12]. Strategies aiming at inhibiting TLR4 signaling pathways have displayed a profound reduction in hepatic fibrogenesis [13]. These results demonstrated the causal roles of TLR4 and TGF-*β*1 signaling in regulating HSC activation and promoting the progression of liver fibrosis.

As the clinically proven antifibrotic therapy is still dependent on the underlying etiology [14], development of novel therapeutic agents suitable for human remains an urgent goal. Andrographolide (Andro), one of the diterpenoids, is purified from the aerial parts of plants of the genus *Andrographis* [15]. In addition to the long history of Andro application in the treatment of inflammatory disorders, it has been recently reported that Andro suppressed the hepatic inflammations and angiogenesis in the thioacetamide-induced fibrotic mice [16]. Meanwhile, Andro also attenuated hepatic apoptosis through modulation of the cannabinoid receptors in fibrotic rat induced by bile duct ligation [17]. Nevertheless, the exact mechanism by which Andro ameliorates liver fibrosis is still unclear. In the present study, we aimed at investigating the effects of Andro on the carbon tetrachloride- (CCl_4_-) induced liver fibrosis in mice and determining whether Andro exerts antifibrosis effects through suppression of inflammation. Our results demonstrated that Andro ameliorated both liver inflammation and fibrosis at least in part through inhibiting the activation of the TLR4/NF-*κ*B and TGF-*β*1/Smad2 signaling pathways in HSC.

## 2. Materials and Methods

### 2.1. Reagents

Andrographolide, carbon tetrachloride (CCl_4_), and dimethyl sulfoxide (DMSO) were purchased from Sigma (St. Louis, CA). Dulbecco's modified Eagle's medium (DMEM), fetal bovine serum (FBS), penicillin/streptomycin solution, 0.05% Trypsin-EDTA, phosphate-buffered saline (PBS), and TRIzol reagent were from Invitrogen Life Technologies (Carlsbad, CA, USA). Anti-NF-*κ*B p-p50 antibody was from Abcam (Cambridge, UK). Anti-*α*-SMA, anti-CD68, and anti-GAPDH antibodies were from Boster Biological Technology Co. Ltd. (Wuhan, China). Anti-TGF-*β*1, anti-p-Smad2, anti-pSmad3, and anti-Smad2/3 antibodies were from Cell Signaling Technology Inc. (Beverly, MA, USA). Anti-NF-*κ*B-p50 antibody was from Santa Cruz Biotechnology Inc. (Santa Cruz, CA, USA). Anti-TLR4 and anti-Smad7 antibodies were from Proteintech Group Inc. (Proteintech, Rosemont, USA). Alexa Fluor 488 and 594-conjugated secondary antibodies were from Invitrogen Life Technologies (Carlsbad, CA, USA). Horseradish peroxidase- (HRP-) conjugated AffiniPure goat anti-mouse IgG and anti-rabbit IgG were purchased from Zhongshan Golden Bridge Biotechnology Co. Ltd. (Beijing, China). Enhanced chemiluminescence (ECL) substrate for the detection of HRP and Protease Inhibitor Cocktail Kit were obtained from Pierce Thermo Scientific (Rockford, USA). Enzyme-linked immunosorbent assay (ELISA) kits for IL-1*β* and IL-6 were purchased from Cusabio Biotech Co. (Wuhan, China). Alanine aminotransferase (ALT), aspartate aminotransferase (AST), and hydroxyproline assay kits were purchased from Nanjing Jiancheng Bioengineering Institute (Nanjing, China).

### 2.2. Animals

Male C57BL/6 mice were obtained from the Center of Experimental Animal of Sun Yat-sen University. All experiments and animal care were approved by the National Institutes of Health Guide for the Care and Use of Laboratory Animals and approved by the Ethics Committee on the Care and Use of Laboratory Animals in Guangdong Pharmaceutical University (Guangzhou, China).

### 2.3. Induction of Hepatic Fibrosis by CCl_4_

Liver fibrosis was induced in 12 C57BL/6 mice by intraperitoneal injection of carbon tetrachloride (CCl_4_) for 6 weeks (0.5 mL/kg body weight, diluted in corn oil, twice a week). Corn oil injection was used as a vehicle control.

### 2.4. Treatment of Hepatic Fibrosis

In the Andro treatment group (*n* = 6), mice were intraperitoneally injected (5 mg/kg) with Andro following each CCl_4_ injection. The same volume of saline was given to the mice in both the normal control group (*n* = 6) and the CCl_4_-induced alone group (*n* = 6). Additionally, one normal control group (*n* = 6) treated with Andro (5 mg/kg) was set to assess the toxic effects of Andro.

### 2.5. Serum Biochemistry

Serum levels of alanine aminotransferase (ALT) and aspartate aminotransferase (AST) were measured using standard enzymatic procedures according to the manufacturer's instruction.

### 2.6. Cell Culture and Treatment

The well-characterized cell line derived from human HSC, LX-2, and also primary HSC were used in in vitro studies. LX-2 was generously provided by Professor Qi Zhang (the Third Affiliated Hospital of Sun Yat-sen University, Guangzhou 510630, China). Cells were cultured in DMEM supplemented with 10% fetal bovine serum and 1% penicillin-streptomycin in a 5% CO2 incubator at 37°C. Cells were synchronized in serum-free DMEM for 24 h followed by Andro administration for additional 24 h with different concentrations (5, 10, and 20 *μ*M).

### 2.7. Western Blot Analysis

Equal amounts of total proteins (30 *μ*g) were fractionated by SDS-PAGE and then transferred onto polyvinylidene difluoride (PVDF) membranes (Millipore Corp., Bedford, MA, USA). After being blocked with 5% nonfat milk in Tris-buffered saline with Tween-20 for 1 h at room temperature, the membranes were incubated with primary antibodies at 4°C overnight and then incubated with the respective secondary antibodies (1 : 5000 dilutions of each antibody) for 1 h at room temperature. Enhanced chemiluminescence detection reagents (Millipore, USA) were used, and the bands were captured using chemiluminescence system (New Life Science Products, Boston, MA, USA).

### 2.8. Immunohistochemistry and Immunofluorescent Staining

Liver specimens fixed in 10% buffered formalin were embedded in paraffin blocks. Liver sections (4 *μ*m thick) were processed using a standard immunostaining protocol. For immunohistochemical analyses, liver sections were separated, rehydrated, and sequentially incubated with primary antibodies and secondary antibodies. The area of positive staining was measured in high-power (×20) fields on each slide and quantified using ImageJ software.

For immunofluorescent staining, the livers were collected and fixed in 10% PBS-buffered formalin for 24 h. The fixed samples were sequentially exposed to 10% and 30% sucrose in PBS for 10 h each and then embedded in Tissue Tek OTC compound (Sakura Finetek, Torrance, CA). The liver sections were permeabilized by 0.25% Triton X-100 and incubated with primary antibody (1 : 100) overnight at 4°C. Then, the liver sections were incubated with corresponding Alexa Fluor 488 and 594-conjugated secondary antibodies (1 : 500) for 1 h at room temperature and stained with DAPI (1 *μ*g/mL) for 10 min. Finally, the sections were captured by an Olympus BX51 microscope (Olympus Co., Tokyo, Japan). The immunofluorescent analyses for LX-2 were conducted in a similar way as described above.

### 2.9. Quantitative Real-Time RT-PCR

Total RNA was extracted from mouse liver tissues, LX-2, or primary HSC using TRIzol reagent followed by treatment with RNase-free DNase (Takara, Dalian, China) for 30 min at 37°C. RNA was reverse-transcribed using a first-strand cDNA kit (Takara, Dalian, China) according to the manufacturer's protocol. RT-PCR was conducted using the PrimeScript RT-PCR Kit (Takara, Dalian, China) according to the manufacturer's instructions. The PCR was run on a StepOnePlus Real-Time PCR System (Applied Biosystems, Foster City, CA, USA). The PCR reactions were carried out at 95°C for 30 s, and 40 cycles 95°C for 5 s and 60°C for 34 s. The relative abundance of the target genes was obtained by calculating against the standard curve and normalized to GAPDH RNA as internal controls. Sequences of PCR primers are summarized in Table 1.

### 2.10. Statistical Analysis

All experiments were performed in at least triplicate, and the results are expressed as the mean ± standard deviation (SD).

Statistical differences between the two groups were analyzed by unpaired Student's *t*-test, and differences between multiple groups of data were analyzed by one-way ANOVA with Bonferroni's correction (GraphPad Prism 5.0). *p* < 0.05 was considered statistically significant.

## 3. Result

### 3.1. Andro Attenuated Liver Fibrosis in Mice after CCl_4_ Induction

**Figure 1:**
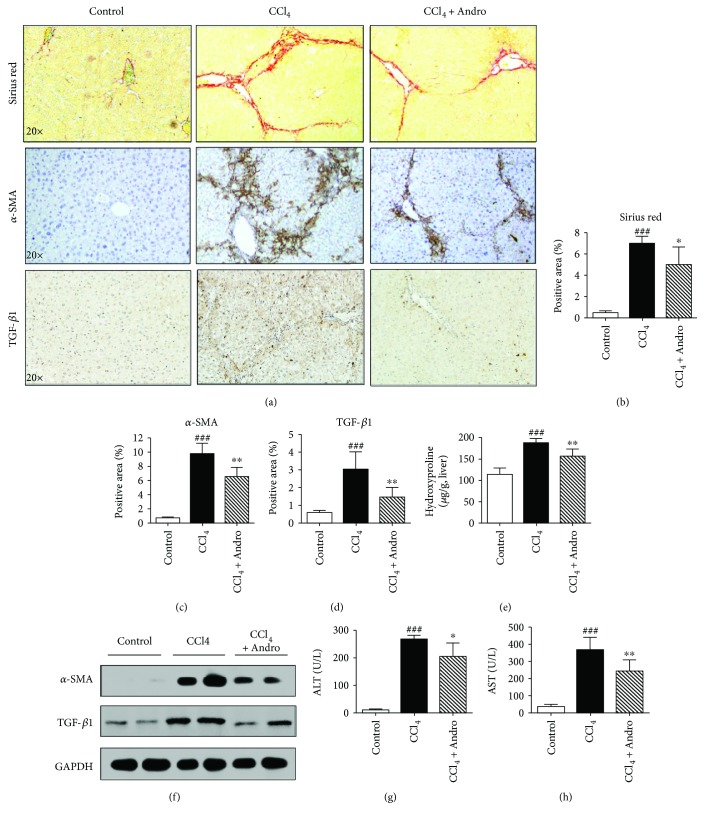
Andrographolide (Andro) improved CCl_4_-induced liver fibrosis in mice. (a) Representative histology of Sirius Red and immunohistochemical staining of *α*-SMA and TGF-*β*1. (b–d) Quantification of positive staining areas was measured by ImageJ software. (e) Hepatic hydroxyproline content. (f) The protein expression of *α*-SMA and TGF-*β*1 was examined by Western blot. (g, h) Serum levels of ALT and AST. *n* = 6. ^###^ *p* < 0.001 versus control mice. ^∗^ *p* < 0.05 and ^∗∗^ *p* < 0.01 versus mice induced by CCl_4_.

To investigate the effects of Andro on liver fibrosis, mice were induced by CCl_4_ for 6 weeks. Both Sirius red staining and hydroxyproline assay showed that collagen accumulation was dramatically increased in CCl_4_-induced mouse liver; however, Andro treatment significantly decreased collagen deposition (Figures 1(a), 1(b), and 1(e)). Furthermore, Andro treatment significantly reduced hepatic expression of *α*-SMA, a marker of activated HSC (Figures 1(a) and 1(c)). In addition, Western blot assays also showed that Andro treatment significantly reduced the protein of *α*-SMA in fibrotic livers induced by CCl_4_ (Figure 1(f)). Meanwhile, TGF-*β*1 was also downregulated by Andro treatment in mice induced by CCl_4_ (Figures 1(a), 1(d), and 1(f)). These results suggest that Andro attenuates the transdifferentiation of HSC into myofibroblasts. Additionally, the elevation of serum ALT and AST induced by CCl_4_ was reduced by Andro treatment (Figures 1(g) and 1(h)), suggesting that Andro also ameliorated CCl_4_-induced chronic liver injury. In addition, we also assessed the side effects of Andro (5 mg/kg); there seemed no toxic effects of Andro on the healthy mice according to the results of H&E staining of the major organs (Figure S3).

### 3.2. Andro Attenuated Liver Inflammation in Mice Induced by CCl_4_

**Figure 2:**
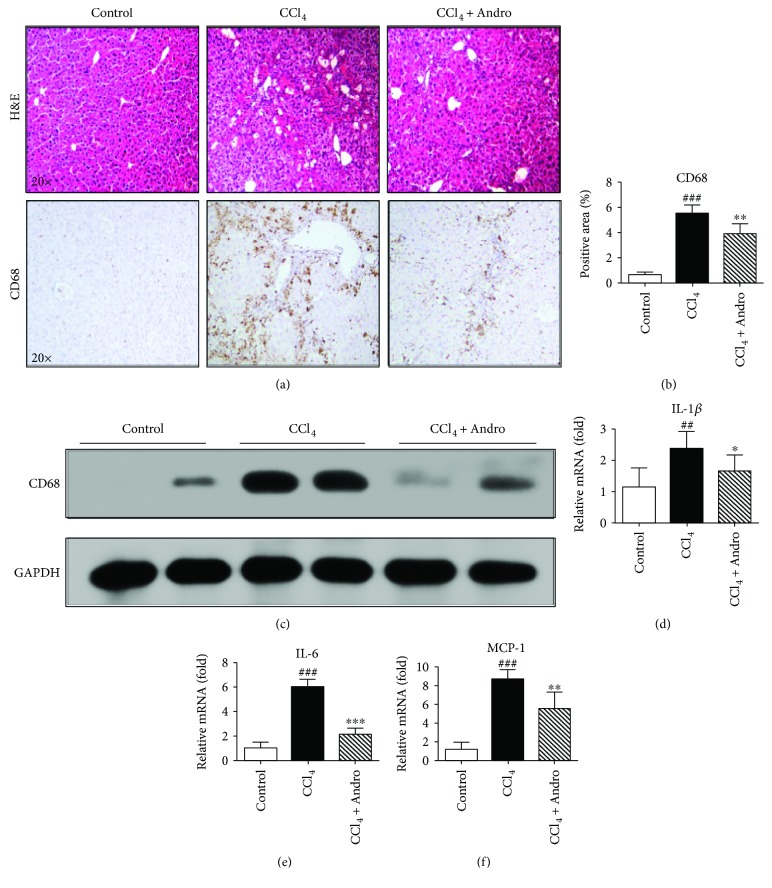
Andro attenuated hepatic inflammation in mice induced by CCl_4_. (a) Representative histology of H&E and immunohistochemical staining of CD68. (b) Quantification of CD68-positive staining areas was measured by ImageJ software. (c) The protein expression of CD68 was examined by Western blot. (d–f) The mRNA levels of IL-1*β*, IL-6, and MCP-1 were measured by q-PCR. *n* = 6. ^##^ *p* < 0.01 and ^###^ *p* < 0.001 versus control mice. ^∗^ *p* < 0.05, ^∗∗^ *p* < 0.01, and ^∗∗∗^ *p* < 0.001 versus mice induced by CCl_4_.

To examine the roles of Andro in chronic liver inflammation, hepatic macrophage infiltration and inflammatory genes were analyzed in CCl_4_-induced mice. H&E staining showed that inflammatory cell infiltration was observed in CCl_4_-induced mouse liver. However, Andro treatment significantly attenuated CCl_4_-induced inflammatory cell infiltration (Figure 2(a)). Meanwhile, the expression of CD68, the macrophage marker, was significantly decreased in mice treated by Andro, as determined by immunochemical staining and Western blotting (Figures 2(a)–2(c)). Furthermore, q-PCR assay showed that hepatic mRNA levels of inflammatory genes, IL-1*β*, IL-6, and MCP-1, were also markedly increased in CCl_4_-induced mice, but significantly decreased in mice administrated by Andro (Figures 2(d)–2(f)). Lastly, as measured by ELISA, the levels of IL-1*β* and IL-6 in the fibrotic liver tissues were also suppressed by Andro (Figure S2).

### 3.3. Andro Inhibited TLR4/NF-*κ*B Signaling Pathway and Reduced Inflammation in CCl_4_ Mice

**Figure 3:**
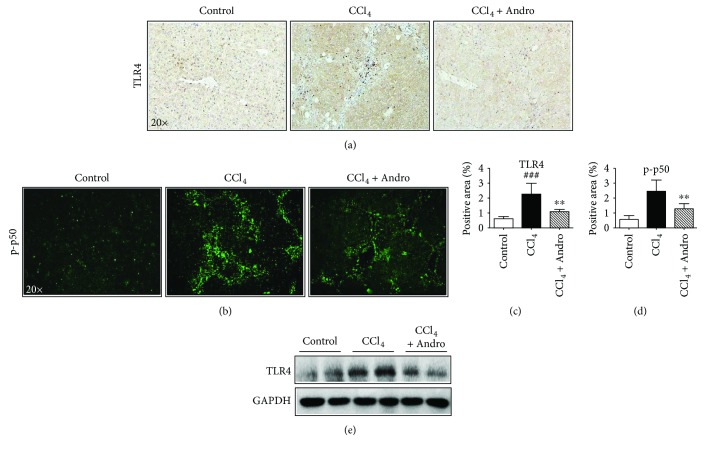
Andro reduced inflammation in CCl_4_-induced mice through inhibition of the TLR4/NF-*κ*B signaling pathway. (a) Representative immunohistochemical staining of TLR4. (b) Representative immunofluorescent staining of NF-*κ*B p-p50. (c, d) Quantification of positive staining areas was measured by ImageJ software. (e) The protein expression of TLR4 was examined by Western blot. *n* = 6. ^###^ *p* < 0.001 versus control mice. ^∗∗^ *p* < 0.01 versus mice induced by CCl_4_.

TLR4 plays a vital role in the inflammatory response during liver fibrosis, which can upregulate the profibrogenic and proinflammatory cytokines through the activation of NF-*κ*B [12]. As shown by the results from immunochemical staining and Western blot, the increased hepatic expression of TLR4 in CCl_4_-induced mice was attenuated by Andro treatment (Figures 3(a), 3(c), and 3(e)). NF-*κ*B p50 is downstream of TLR4 and involved in the liver fibrosis [18]. And Andro is also a specific inhibitor of p50 [19]. As shown in Figures 3(b) and 3(d), Andro significantly reduced p50 expression in mice induced by CCl_4_. These data demonstrated a direct correlation between the anti-inflammatory effects of Andro and the inhibition of the TLR4/NF-*κ*B p50 signaling pathway.

### 3.4. Andro Inhibited TGF-*β*1/Smad2 Signaling and Attenuated HSC Activation

**Figure 4:**
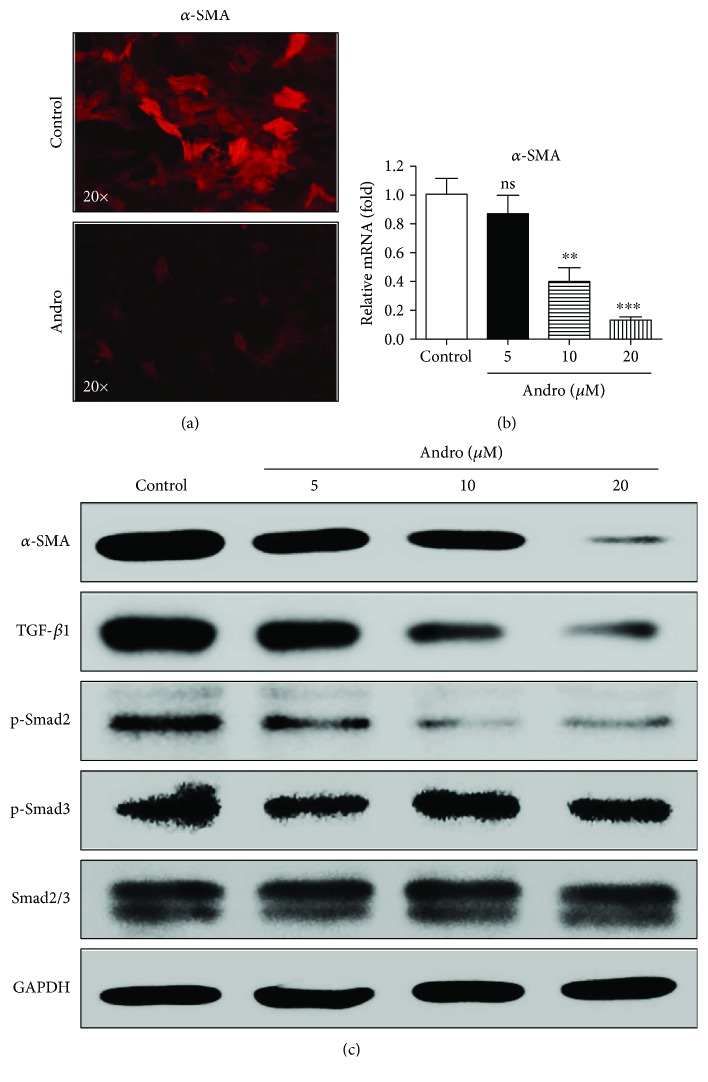
Andro attenuated HSC activation through inhibition of the TGF-*β*1/Smad2 signaling pathway. (a) Representative immunofluorescent staining of *α*-SMA. (b) The mRNA levels of *α*-SMA were measured by q-PCR. (c) The protein expression of *α*-SMA, TGF-*β*1, p-Smad2, p-Smad3, and Smad2/3 was examined by Western blot. *n* = 3. ^∗∗^ *p* < 0.01 and ^∗∗∗^ *p* < 0.001 versus control.

TGF-*β*1/Smad signaling is involved in the activation of HSC during liver fibrogenesis [20]. The marker of activated HSC, *α*-SMA, was downregulated by Andro in both LX-2 cell line and primary HSC (Figures 4(a)–4(c), Figure S1A). Western blotting showed that TGF-*β*1 and p-Smad2 in HSC were downregulated by Andro in a dose-dependent manner (Figure 4(c)). However, there was no change in the phosphorylation of Smad3 (Figure 4(c)), indicating that Andro suppressed HSC activation probably by blocking TGF-*β*1/Smad2 signaling. Meanwhile, we found that Andro showed no influence on the expression of Smad7 (Figure S4), an inhibitory Smad that could block the overactivation of TGF-*β*1 signals via inhibition of Smad2/3 [21, 22]. This result indicated that the downregulation of TGF-*β*1/Smad2 by Andro may have no relationship with Smad7.

### 3.5. Andro Suppressed the TLR4/NF-*κ*B p50 Signaling in HSC and Reduced the Proinflammatory Chemokines

**Figure 5:**
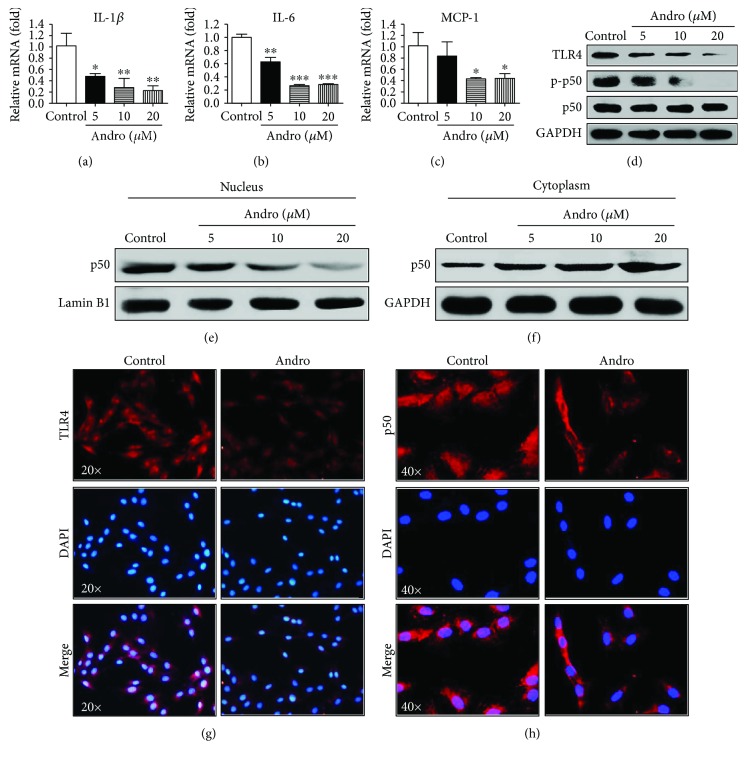
Andro suppressed proinflammatory chemokines mediated by TLR4/NF-*κ*B p50 signaling in HSC. (a–c) The mRNA levels of IL-1*β*, IL-6, and MCP-1 were measured by q-PCR. (d) The protein expression of TLR4, p-p50, and p50 was examined by Western blot. (e, f) p50 levels in the cytosol and nucleus were assayed. (g) Representative immunofluorescent staining of TLR4. (h) Nuclear translocation of NF-*κ*B p50 in HSC was assayed by immunofluorescence. *n* = 3. ^∗^ *p* < 0.05, ^∗∗^ *p* < 0.01, and ^∗∗∗^ *p* < 0.001 versus control.

We next measured the effects of Andro on the expression of proinflammatory cytokines IL-1*β*, IL-6, and MCP-1 in HSC. As depicted in Figures 5(a)–5(c), q-PCR assay showed that the expression levels of cytokines in LX2 cells were significantly reduced by Andro compared with the vehicle control. These results were well consistent with the findings in the primary activated HSC isolated from the fibrotic mice (Figure S1D–F). The TLR pathway is mainly involved in the upregulation of intrahepatic inflammatory cells and HSC activation after hepatic injury [9, 23]. Consistent with the anti-inflammatory features in HSC, Andro also suppressed the expression of TLR4 as assayed by Western blotting, immunofluorescent staining, and q-PCR assay (Figures 5(d) and 5(g), Figure S1C).

Treatment with Andro significantly abrogated the phosphorylation of NF-*κ*B p50 in LX2 cells (Figure 5(d)). In addition, immunofluorescent staining was conducted to tract p50 translocation to further investigate the effect of Andro on the NF-*κ*B signaling pathway. As illustrated in Figure 5(h), the majority of p50 was located in the nucleus in culture-activated HSC. After Andro treatment for 2 h, the majority of p50 was located in the cytoplasm, suggesting Andro inhibited p50 nuclear translocation. This finding was also proved by the Western blot analysis on nuclear and cytoplasm extracts of LX2 treated or not with Andro (Figures 5(e) and 5(f)). Taken together, these results demonstrated that Andro specifically blocked the TLR4/NF-*κ*B p50 pathway, contributing to the suppression of intrahepatic inflammation.

## 4. Discussion

Liver fibrosis results from acute or chronic liver injury, characterized by tissue repair with a concomitant inflammatory response and ECM accumulation [24]. The present study aimed to elucidate the effects and potential mechanism of Andro on the CCl_4_-induced liver fibrosis. Our results showed that Andro protected against chronic liver inflammation and fibrosis, which was linked with the inhibition of the TLR4/NF-*κ*B p50 and TGF-*β*1/Smad2 signaling pathways.

Activation of HSC is recognized as a critical marker of hepatic fibrosis, represented by the upregulation of *α*-SMA and collagen [25]. In our current study, we demonstrated that Andro markedly suppressed the activation of HSC and reduced the accumulation of collagen in mice with liver fibrosis. This finding was in line with a recent report that Andro treatment resulted in a significant decrease in hepatic fibrogenesis and *α*-SMA upregulation [16]. Additionally, we found that serum markers of liver damage, including ALT and AST, were also attenuated by Andro (5 mg/kg) in mice induced by CCl_4_. And also, we assessed the side effects of Andro (5 mg/kg) by H&E staining of the major organs and found no toxic effects of Andro on the healthy mice (Figure S3). However, higher concentrations of Andro (50 mg/kg) aggravated the hepatic injury and sharply decreased the body weights of the mice (data not shown), suggesting that a rational dose range of Andro should be considered in the treatment of liver fibrosis.

Among the various profibrogenic mediators, TGF-*β*1 is the most effective one involved in hepatic fibrogenesis influencing the activation of HSC [26]. The TGF-*β* family composes of three closely related isoforms (TGF-*β*1, TGF-*β*2, and TGF-*β*3) that usually have similar bioactivities *in vitro*, while triggering distinctive biological responses *in vivo* [5]. In the form of secreted peptide, TGF-*β*1 exhibits its biological activities through the Smad-dependent or independent pathways. The abrogation of TGF-*β*1 signaling through both Smad2 and Smad3 has been verified to be able to decrease liver fibrosis [8, 27]. In the current study, we provided evidence that Andro downregulated the expression of TGF-*β*1 and the phosphorylation of Smad2 in LX-2 cell line. Interestingly, the level of phosphorylated Smad3 was not affected by Andro. Our results showed the relationship between the Andro-mediated inactivation of HSC and the TGF-*β*1/Smad2 pathway. In view of the differential roles of Smad2 and Smad3 in hepatic fibrosis, Uemura et al. verified that Smad3 is more implicated than Smad2 in the morphological and functional maturation of myofibroblasts by transfecting HSC with adenoviruses expressing wild-type and dominant-negative Smad2 or Smad3 [28], whereas recent study from Koo et al. indicated that endoplasmic reticulum (ER) stress in HSC promoted hepatic fibrosis by inducing overexpression of Smad2. They showed that the levels of Smad2, but not Smad3, were increased in fibrotic liver tissues from patients or mice under ER stress and knockdown of Smad2 reduced ER stress-mediated activation of HSC [29]. However, our previous work presented that inhibition of Robo1 attenuated hepatic fibrosis through downregulation of the phosphorylation of both Smad2 and Smad3 in HSC independent of TGF-*β*1 [25]. Thus, further work is still warranted to determine the precise mechanism by which Smad2 and Smad3 affect hepatic fibrogenesis. In opposite to Smad2/3, Smad7 serves as an inhibitory Smad that could block the activation of TGF-*β*1 signals via inhibition of Smad2/3, playing a protective role in liver fibrosis [21, 22]. Unexpectedly, we found no influence of Andro on the expression of Smad7 (Figure S4). In this context, Andro downregulated the TGF-*β*1/Smad2 pathway independent of Smad7, suggesting that Smad7 was not involved in the antifibrotic property of Andro.

Besides, inflammation is also considered another key mediator of the activation of HSC and the pathogenesis of hepatic fibrosis [30]. Increasing evidences showed a pivotal role for Andro in effectively controlling the inflammatory activity in various diseases of the lung, kidney, and colon [31–33]. In the current study, Andro is capable of suppressing inflammatory response both in HSC and in CCl_4_-induced fibrotic mice. During liver injury, the macrophage has been presumed to be the major source of secretory inflammatory factors which activate the HSC and aggravate hepatic fibrosis [34]. Consistent with decreased inflammatory cell infiltration assayed by H&E staining, we also found that CD68, a marker of macrophage, was significantly suppressed by Andro treatment. Accordingly, Andro also dramatically downregulated the levels of inflammatory factors (IL-1*β*, IL-6, and MCP-1) both *in vitro* and *in vivo*. These results demonstrated that Andro prevented against liver injury and fibrosis through control of inflammation.

TLR4, responsible for detecting LPS from Gram-negative bacteria, is a pattern recognition receptor belonging to the TLR family that modulates innate immunity [35]. Numerous evidences have suggested the corroborative role of the TLR4/NF-*κ*B pathway in the pathophysiological process of inflammation and liver fibrogenesis. TLR4 is capable of triggering the rapid activation of its downstream signaling, NF-*κ*B, which upregulates the production of proinflammatory cytokines such as IL-1*β* and IL-6 [36]. TLR4 deficiency resulted in less fibrosis in CCl_4_ or bile duct ligation- (BDL-) induced fibrotic mice [9]. Furthermore, a nucleotide variation in the TLR4 gene brought about protection against the progression of liver fibrosis in humans [37]. In our work, we found that the decreased level of TLR4 expression was accompanied by suppression of the synthesis of proinflammatory cytokines, including IL-1*β*, IL-6, and MCP-1 both *in vitro* and *in vivo*. In light of the downstream signaling of TLR4, we also provided evidence that Andro markedly inhibited the NF-*κ*B signaling. *In vitro*, Andro reduced the phosphorylation and nuclear translocation of NF-*κ*B p50 in LX-2 cell line, while *in vivo*, administration of Andro significantly reduced the phosphorylated NF-*κ*B p50 in the fibrotic livers. These results demonstrated that Andro improved inflammation response partly by suppression of the TLR4/NF-*κ*B signaling pathway during liver fibrogenesis. Given that TLR4 enhances TGF-*β*1 signaling in hepatic fibrosis [9] and Andro downregulates the expression of TGF-*β*1, we suppose that the protection of Andro against liver fibrosis has a direct correlation with shutting down the TLR4 and TGF-*β*1 signaling pathways.

Apart from inflammation, the involvement of oxidative stress and reactive oxygen species (ROS) in the liver fibrogenesis of various experimental models has been definitely confirmed in many previous reports [38]. Many natural compounds exert their beneficial effects, at least in part, by antioxidant properties [39]. Thus, in the present work, we also performed the measurement of MDA to assess the antioxidant ability of Andro on liver fibrosis. Unfortunately, there was no significant difference in the MDA levels between the CCl_4_ groups with or without Andro treatment (Figure S5B). This result indicated that in the situation of hepatic fibrosis, the antioxidant properties of Andro were relatively limited.

Despite the significant findings revealed by this investigation, limitations still exist. In our present work, Andro exerts antifibrosis effects through the suppression of inflammation. However, the antifibrosis effects of Andro herein are prophylactic but not therapeutic as the administration of Andro is after each of the CCl_4_ injections in our study. Hence, for a better understanding of the therapeutic antifibrosis potential of Andro, further work is needed in mice that have already developed chronic fibrosis after 6 weeks of CCl_4_ treatment. This will be taken into consideration in our future study investigating the effects of Andro on advanced liver fibrosis.

## 5. Conclusions

In conclusion, our current study demonstrated that Andro ameliorated liver fibrosis in part through suppressing the activation of the TLR4/NF-*κ*B and TGF-*β*1/Smad2 signaling pathways. It is promising to develop Andro as a potential therapeutic candidate for the treatment of hepatic fibrosis.

## Figures and Tables

**Table 1:** Primer sequences for real-time PCR.

Genes	Forward primer (5′-3′)	Reverse primer (5′-3′)
Mouse		
*α*-SMA	GTTCAGTGGTGCCTCTGTCA	ACTGGGACGACATGGAAAAG
IL-1*β*	TGCCACCTTTTGACAGTGATG	ATGTGCTGCTGCGAGATTTG
IL-6	ACCAGAGGAAATTTTCAATAGGC	TGATGCACTTGCAGAAAACA
MCP-1	ATTGGGATCATCTTGCTGGT	CCTGCTGTTCACAGTTGCC
GAPDH	AGGAGTAAGAAACCCTGGAC	CTGGGATGGAATTGTGAG
Human	CCAGAGCCATTGTCACACAC	CAGCCAAGCACTGTCAGG
*α*-SMA	TTCGACACATGGGATAACGAGG	TTTTTGCTGTGAGTCCCGGAG
IL-1*β*	ACTCACCTCTTCAGAACGAATTG	
IL-6	CCATCTTTGGAAGGTTCAGGTTG	TGGAATCCTGAACCCACTTCT
MCP-1	CAGCCAGATGCAATCAATGCC	GCCATCACGCCACAGTTTC
GAPDH	ACAACTTTGGTATCGTGGAAGG	

## Abbreviations

*α*-SMA: *α*-Smooth muscle actin
HSC: Hepatic stellate cells
ECM: Extracellular matrix
Andro: Andrographolide
CCl_4_: Carbon tetrachloride
ALT: Alanine aminotransferase
AST: Aspartate aminotransferase
TGF-*β*1: Transforming growth factor-*β*1
TLR4: Toll-like receptor-4
NF-*κ*B: Nuclear factor *κ*B.

## Supplementary Materials

Supplementary Materials Figure S1: effects of Andro on profibrotic and proinflammatory factors in primary HSC. The mRNA levels of *α*-SMA, TGF-*β*1, TLR4, IL-1*β*, IL-6, and MCP-1 were measured by q-PCR. *n* = 3; ^∗^ *p* < 0.05, ^∗∗^ *p* < 0.01, and ^∗∗∗^ *p* < 0.001 versus control. Figure S2: effects of Andro on proinflammatory cytokines in liver tissues. The levels of IL-1*β* and IL-6 of liver tissues were measured by ELISA. *n* = 6; ^###^ *p* < 0.001 versus control mice; ^∗∗^ *p* < 0.01 and ^∗∗∗^ *p* < 0.001 versus mice induced by CCl_4_. Figure S3: toxicity assessment of Andro by H&E staining of the major organs. Figure S4: the protein expression of Smad7 was examined by Western blot. Figure S5: (A) molecular structure of Andro. (B) Hepatic malondialdehyde (MDA) levels were measured using thiobarbituric acid reactive substance (TBARS) assay. *n* = 6; ^###^ *p* < 0.001 versus control mice. Figure S6: representative immunohistochemical staining of *α*-SMA, TGF-*β*1, CD68, and TLR4.
