# Supplementary material for: Andrographolide Ameliorates Liver Fibrosis in Mice: Involvement of TLR4/NF-κB and TGF-β1/Smad2 Signaling Pathways
Source: Oxid Med Cell Longev. 2018 Mar 18;2018:7808656. doi: 10.1155/2018/7808656 (PMC5878918; doi:10.1155/2018/7808656)
Supplement: Supplementary Materials — Figure S1: effects of Andro on profibrotic and proinflammatory factors in primary HSC. The mRNA levels of α-SMA, TGF-β1, TLR4, IL-1β, IL-6, and MCP-1 were measured by q-PCR. n = 3; ∗ p < 0.05, ∗∗ p < 0.01, and ∗∗∗ p < 0.001 versus control. Figure S2: effects of Andro on proinflammatory cytokines in liver tissues. The levels of IL-1β and IL-6 of liver tissues were measured by ELISA. n = 6; ### p < 0.001 versus control mice; ∗∗ p < 0.01 and ∗∗∗ p < 0.001 versus mice induced by CCl4. Figure S3: toxicity assessment of Andro by H&E staining of the major organs. Figure S4: the protein expression of Smad7 was examined by Western blot. Figure S5: (A) molecular structure of Andro. (B) Hepatic malondialdehyde (MDA) levels were measured using thiobarbituric acid reactive substance (TBARS) assay. n = 6; ### p < 0.001 versus control mice. Figure S6: representative immunohistochemical staining of α-SMA, TGF-β1, CD68, and TLR4. [file 7808656.f1.docx]

**Methods**

**Cell isolation**

Primary HSCs were isolated from fibrotic mice (treated with CCL_4_ for 6 weeks) using a two-step collagenase-pronase perfusion of mouse livers as described (*Hepatology* 2011; **53**: 1730-1741).

**Enzyme-linked immunosorbent assay (ELISA)**

The levels of IL-1βand IL-6 in liver tissues were analyzed using commercial ELISA kits according to the manufacturer’s instructions. Briefly, 100 μL of standard and sample were added into each well and incubated for 2 h at 37 °C. After removing the supernatant, 100 μL of biotin-antibody (1x) was added and incubated for 1 h at 37°C. After three times wash with PBS, 100 μL/per well of HRP-avidin (1x) was added and incubated for 1 h at 37 °C, followed by incubation with 90 μL of TMB substrate for 30 min at 37 °C in dark. After adding 50 μL /well of stop solution, the optical density was measured within 5 min using a microplate reader at 450 nm.

**Hydroxyproline assay**Hepatic hydroxyproline content was measured using commercial hydroxyproline assay kit according to the manufacturer’s instructions. Briefly, liver samples were hydrolyzed at 95 ℃ for 20 min, then adjusted to pH 6.5 and filtered through activated charcoal. After centrifugation, the supernatant was mixed with detecting liquid and incubated at 60 ℃ for 15 min. Last, samples were measured using a microplate reader at 550 nm.

**Hepatic malondialdehyde (MDA) assay**

MDA was measured by TBARS formation to assess the antioxidant properties of Andro on liver fibrosis. Briefly, liver tissues were homogenized in PBS containing 1X BHT. Then the samples were mixed with SDS lysis solution and 0.52 % (pH 3.5) aqueous solution of thiobarbituric acid. After incubation at 95°C for 40 minutes, the samples were centrifuged at 3500 rpm for 10 min. The supernatant was estimated using a microplate reader at 532 nm. The results were expressed as nmol MDA/mg protein.

**Figures**


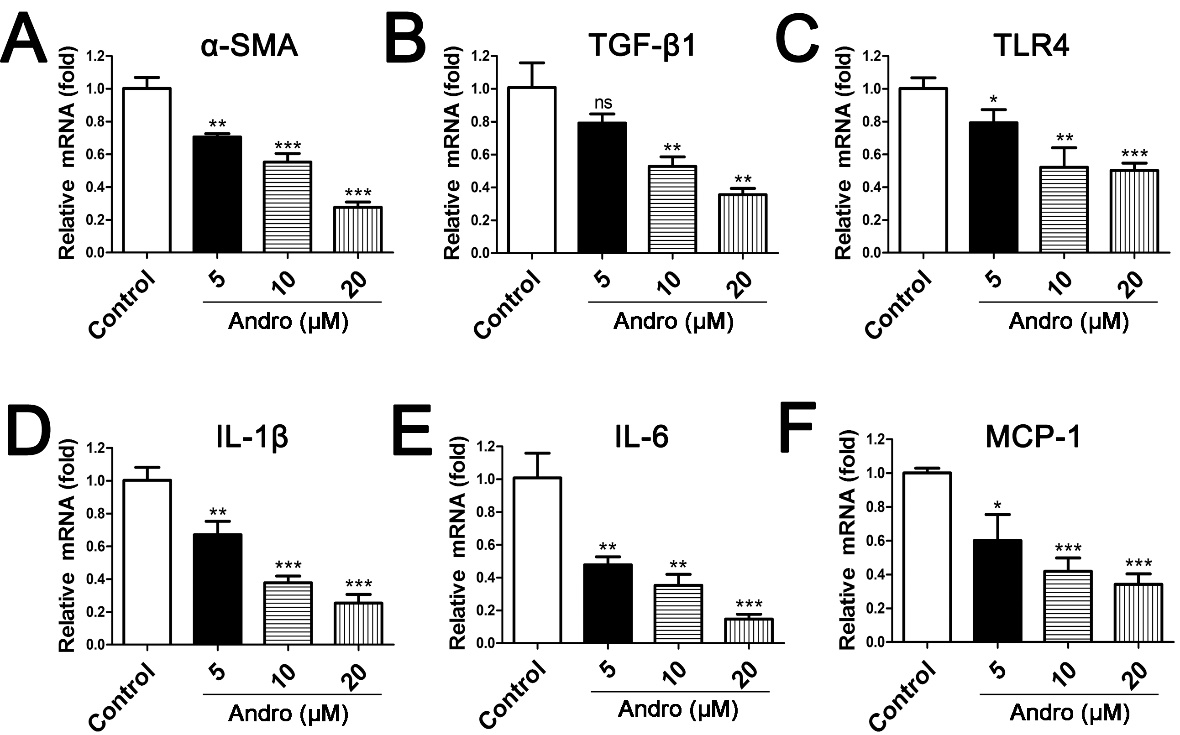


**Fig. S1.** Effects of Andro on pro-fibrotic and pro-inflammatory factors in primary HSCs. The mRNA levels of α-SMA, TGF-β1, TLR4, IL-1β, IL-6, MCP-1 were measured by q-PCR. n=3; ^*^*p* <0.05, ^**^*p* < 0.01, ^***^*p* <0.001 vs Control.


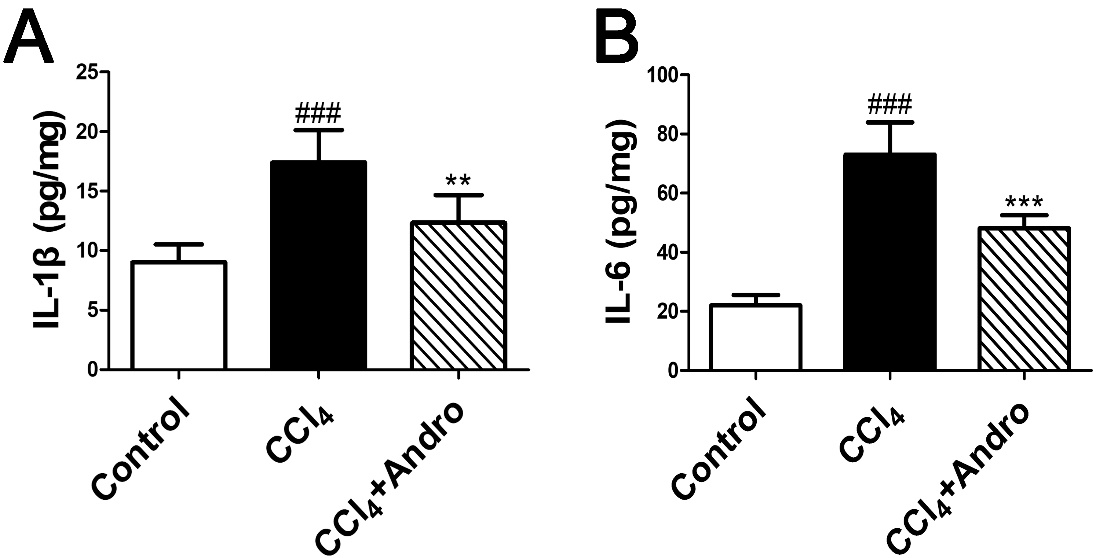


**Fig. S2.** Effects of Andro on pro-inflammatory cytokines in liver tissues. The levels of IL-1β and IL-6 of liver tissues were measured by ELISA. n=6; ###p < 0.001 vs control mice; ^**^*p* < 0.01, ^***^*p* < 0.001 vs mice induced by CCl_4_.


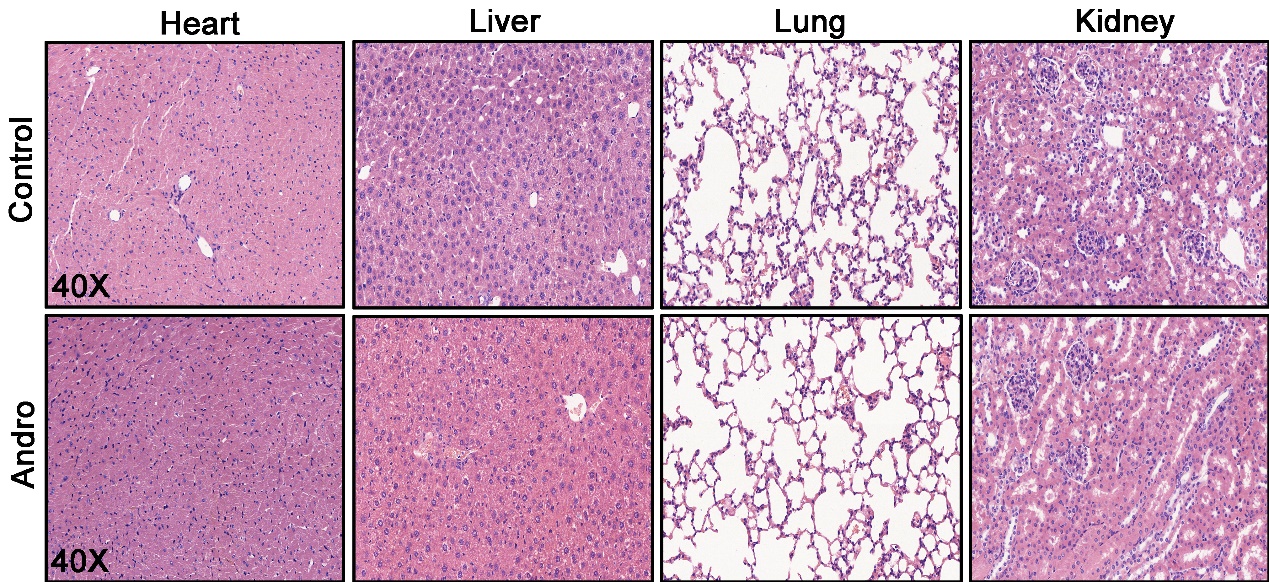


**Fig. S3.** Toxicity assessment of Andro by HE staining of the major organs.


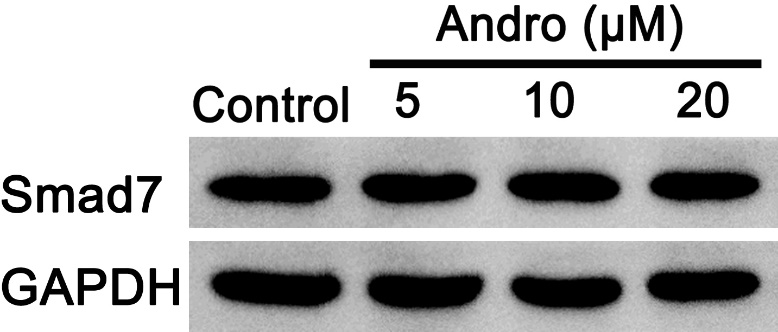


**Fig. S4.** The protein expression of Smad7 was examined by Western blot.


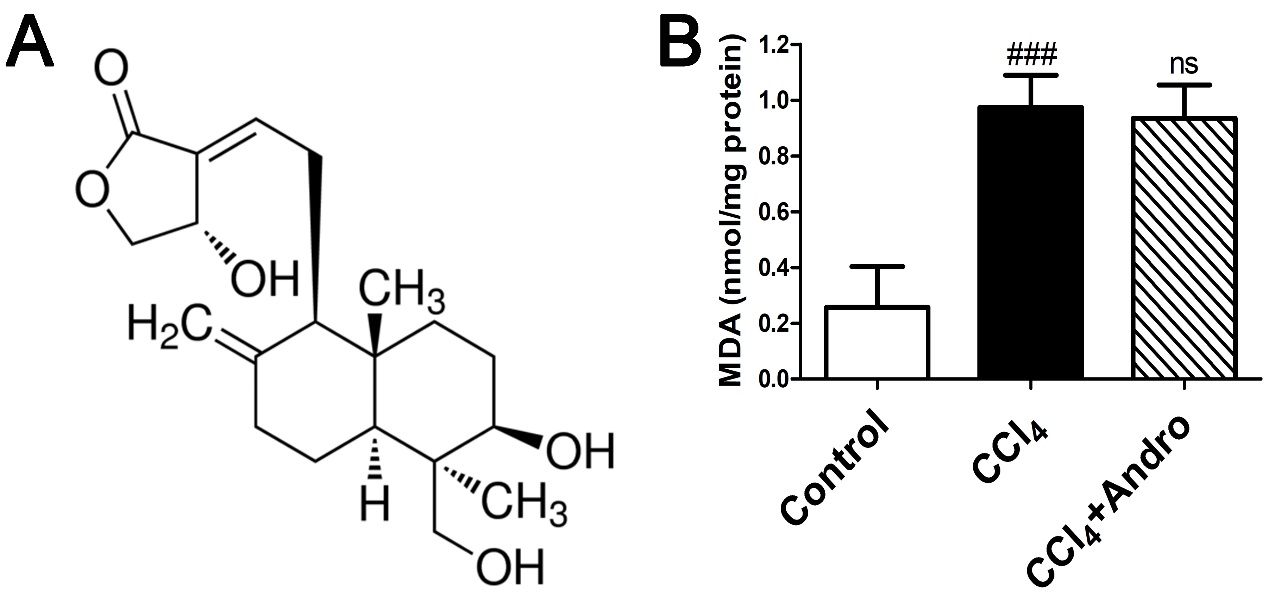


**Fig. S5.** (A) Molecular structure of Andro. (B) Hepatic malondialdehyde (MDA) levels were measured using thiobarbituric acid reactive substances (TBARS) assay. n=6; ###p < 0.001 vs control mice.


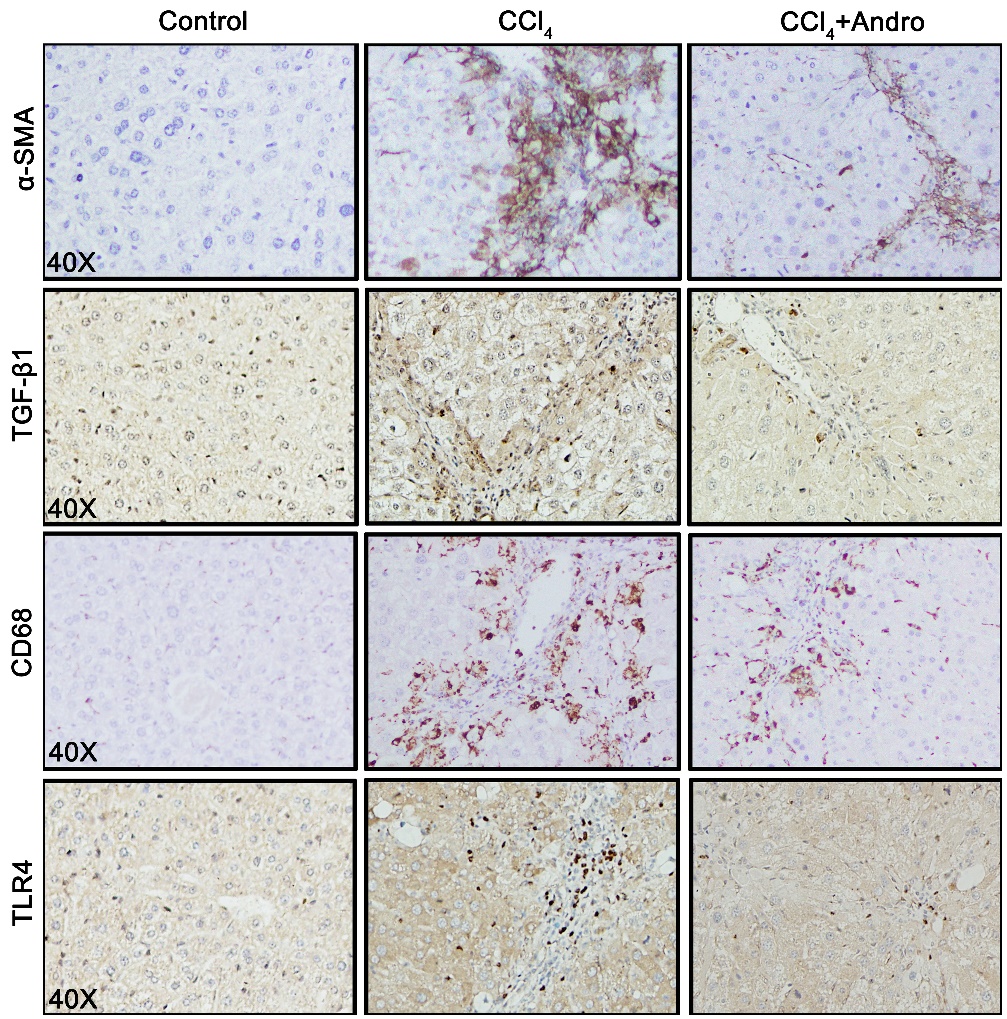


**Fig. S6.** Representative immunohistochemical staining of α-SMA, TGF-β1, CD68 and TLR4.
